# Antibacterial effects of the artificial surface of nanoimprinted moth-eye film

**DOI:** 10.1371/journal.pone.0185366

**Published:** 2017-09-21

**Authors:** Kiyoshi Minoura, Miho Yamada, Takashi Mizoguchi, Toshihiro Kaneko, Kyoko Nishiyama, Mari Ozminskyj, Tetsuo Koshizuka, Ikuo Wada, Tatsuo Suzutani

**Affiliations:** 1 LCD Technology Development Center, Development Group, Display Device Company, Sharp Corporation, Tenri, Nara, Japan; 2 New Business Promotion Center, Development Group, Display Device Company, Sharp Corporation, Tenri, Nara, Japan; 3 Next Generation IGZO Technology Development Center, Development Group, Display Device Company, Sharp Corporation, Tenri, Nara, Japan; 4 Department of Microbiology, Institutes for Biomedical Sciences, Fukushima Medical University School of Medicine, Fukushima, Japan; 5 Department of Cell Science, Institutes for Biomedical Sciences, Fukushima Medical University School of Medicine, Fukushima, Japan; University of Akron, UNITED STATES

## Abstract

The antibacterial effect of a nanostructured film, known as “moth-eye film,” was investigated. The moth-eye film has artificially formed nano-pillars, consisting of hydrophilic resin with urethane acrylate and polyethylene glycol (PEG) derivatives, all over its surface that replicates a moth’s eye. Experiments were performed to compare the moth-eye film with a flat-surfaced film produced from the same materials. The JIS Z2801 film-covering method revealed that the two films produced a decrease in *Staphylococcus aureus* and *Esherichia coli* titers of over 5 and 3 logs, respectively. There was no marked difference in the antibacterial effects of the two surfaces. However, the antibacterial effects were reduced by immersion of the films in water. These results indicated that a soluble component(s) of the resin possessed the antibacterial activity, and this component was identified as PEG derivatives by time-of-flight secondary ion mass spectrometry (TOF-SIMS) and Fourier transform infrared spectroscopy (FT-IR). When a small volume of bacterial suspension was dropped on the films as an airborne droplet model, both films showed antibacterial effects, but that of the moth-eye film was more potent. It was considered that the moth-eye structure allowed the bacteria-loaded droplet to spread and allow greater contact between the bacteria and the film surface, resulting in strong adherence of the bacteria to the film and synergistically enhanced bactericidal activity with chemical components. The antibacterial effect of the moth-eye film has been thus confirmed under a bacterial droplet model, and it appears attractive due to its antibacterial ability, which is considered to result not only from its chemical make-up but also from physical adherence.

## Introduction

Contact infection is thought to be a major route of infection for various skin and mucosa, respiratory and gastro-intestinal infections. Indirect human-to-human infections via various touchable surfaces with which we come into contact during activities of daily living often occur, even in the case of respiratory and gastro-intestinal infections as well as droplet and oral infections. Therefore, the cleaning and disinfection of touchable surfaces in our daily life as well as our hands is important to prevent various infections.

For this reason, antimicrobial surface technology has recently received a good deal of attention. Generally there are two approaches: the first is the chemical approach [1], such as surface polymerization, functionalization and derivatization; and the second is the physical approach in which the surface architecture is modified [2, 3]. Both approaches are mainly aimed at killing bacteria directly or preventing the attachment of bacteria.

As for the chemical approach, resin materials including bactericidal materials, such as silver, quaternary ammonium compounds and fluoride ion, are coated on base films. Silver nanoparticles are well-known for their powerful bactericidal effect, which is realized by increasing the concentration of silver ions with silver particles reduced to nanometer size. Chemical bactericidal surfaces demonstrate antibacterial effects when the bactericidal components are eluted into bacterial solutions as ions after immersion or exposure to bacterial solutions. However, this approach still has several problems to be resolved. There are escalating concerns as to whether the extensive exposure of the bactericidal ingredient to bacteria may actually provoke the development of drug-resistant bacteria, such as nanosilver-resistant microorganisms [4]. It should be noted that the chemical antibacterial effect requires the use of water as an intervening medium as the core elements for killing the bacteria are ions in solutions. If the water volume is insufficient or the water evaporates shortly after bacterial attachment, the antibacterial activity may be inhibited. Moreover, if all the bactericidal ingredients are eluted out, the film would lose its antimicrobial ability.

In the meantime, as for physical approach, micrometer- or nanometer-sized structures formed at the surface produce the antibacterial effect. This approach has attracted the interest of researchers in combination with the increased attention to biomimetic technology. It was found that shark skin [5, 6] and sacred lotus leaves [7] have the ability to prevent bacterial attachment. The wings of dragonflies and cicadas are reported to show antibacterial activity [8–11]. According to the reports, artificial nanostructured surfaces are also reported to have a physical bactericidal effect [10, 12–14]. In addition, nanometer-sized particles formed using n-butyl cyanoacrylate have a similar effect [15], with this material known as a biodegradable medical material used in clinical practice. The merit of a physical approach is that it raises fewer concerns with regard to the development of drug-resistant bacteria. However, the drawback to this approach is that the bactericidal potency is likely to be lower than that of the chemical approach. It should be noted that this approach requires bacteria to come into direct contact with the surface.

The moth-eye film possesses a unique array of nanostructures that exhibit a variety of functions including not only low reflectivity [16, 17], but also super-hydrophobicity and super-hydrophilicity (the nanostructures enhanced intrinsic hydrophobic and hydrophilic properties of the resin following Wenzel’s law [18]), as well as antifouling, antifog [19] and antibacterial [13, 20–21] properties. In this study, we evaluated the antibacterial activity of the moth-eye film based on its total characteristics, including the antibacterial properties possessed by the resin itself and the quick drying properties achieved by the artificially fabricated moth-eye structure.

## Materials and methods

### Films

**Fig 1:**
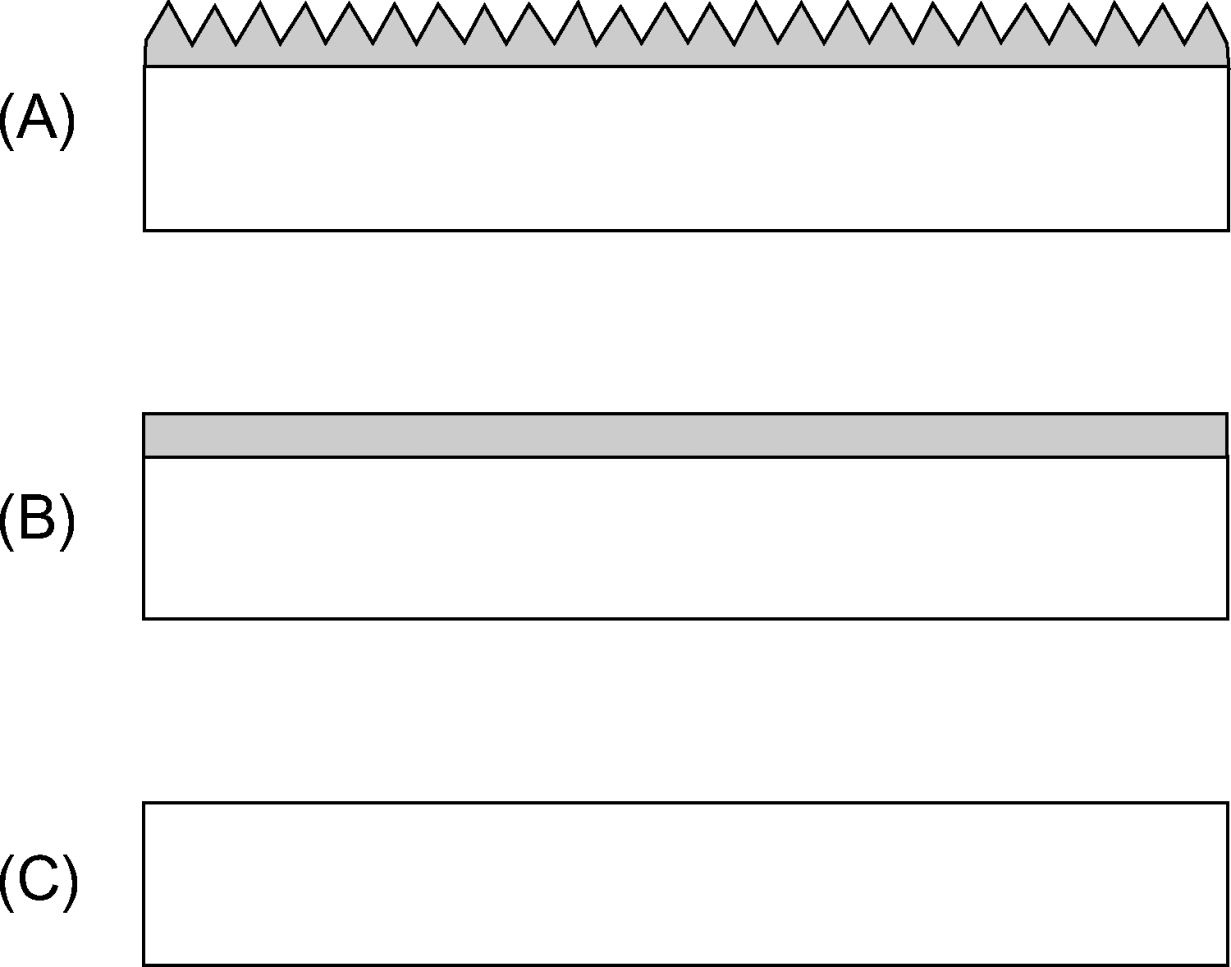
Schematic cross-sectional views of sample films. (A) Sample A; moth-eye film (Moth) formed from hydrophilic urethane acrylate on PET film, (B) Sample B; flat- surfaced film (Flat) formed from the same hydrophilic urethane acrylate as Sample A on PET film, and (C) Sample C; PET film (PET).

We compared three types of sample film: moth-eye (Moth) and flat-surfaced (Flat) films formed from hydrophilic resin (Sample A and B, respectively), which was composed of urethane trifunctional acrylate having polyethylene glycol (PEG) chains as spacers, and polyethylene terephthalate (PET) film (Sample C), which was the base film for Sample A and B (Fig 1). The moth-eye film (Sample A) was fabricated by a nanoimprint method using ultra-violet curable resins, and the pitch and height of the moth-eye structure used in this study were both approximately 200nm.

### Bacterial and fungal strains

*Staphylococcus aureus* (*S*. *aureus*) NBRC12732 strain, a Gram-positive, sphere-shaped bacterium, and Gram-negative rod-shaped bacterium *Esherichia Coli* (*E*. *coli*) NBRC3972 strain were kindly supplied by the National Institute of Technology and Evaluation (Tokyo, Japan).

### Preparation of microbial suspension

After overnight culture of *S*. *aureus* and *E*. *coli* on a nutrient agar medium (NA) plate (Nissui Pharmaceutical Co. Ltd., Tokyo, Japan) at 37°C, one colony was selected, inoculated on a new NA plate and cultured again overnight at 37°C. The bacteria from the second culture were then suspended in 1/500 nutrient broth (1/500 NB; Eiken Chemical Co. Ltd., Tochigi, Japan) at McFarland Standard 1.0, which contained around 10^8^ cfu/ml bacterial cells.

### Antibacterial assay

The antibacterial effects of the films were estimated using two different methods: a film covering method [Japanese Industrial Standards (JIS) Z2801] and a droplet method, which was developed to evaluate the practical effectiveness of the moth-eye film in this study.

#### i) Film covering method (JIS Z2801 method)

**Fig 2:**
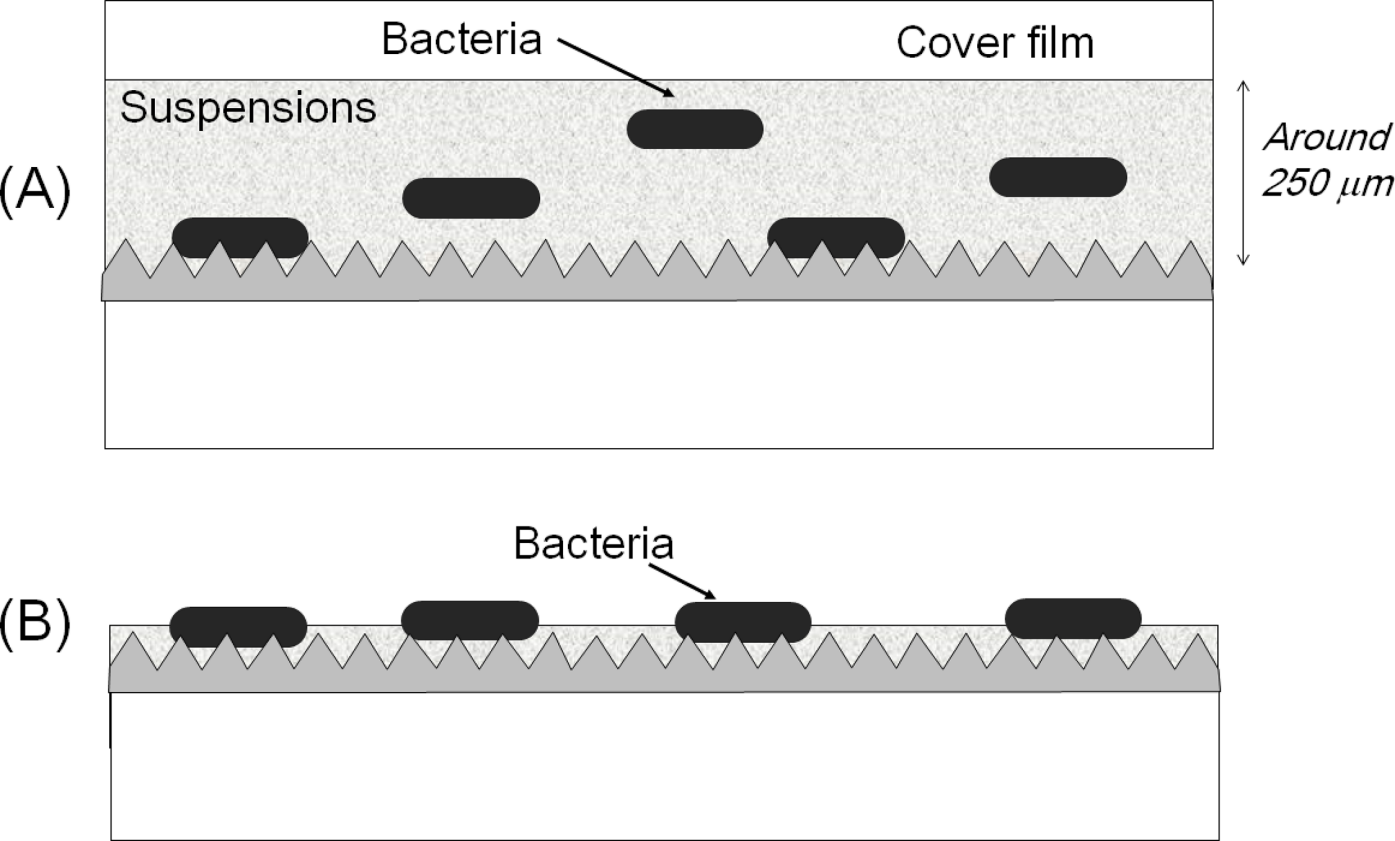
Schematic cross-sectional views of sample films demonstrating the two assays. (A) The film covering method: The bacterial solution was sandwiched between the upper cover film and the lower sample film at a thickness of around 250 μm. The image depicts Sample film A; however, Sample B and C were assayed using the same procedure. (B) The Droplet method: The bacterial solution (less than 10 μm in thickness) was not covered by any film to allow drying.

This assay, based on the ISO 22196 test method, is one of the most widely used antibacterial assays worldwide. This assay has two distinctive features: the use of a covering film to keep the bacteria suspension at a constant thickness of a few hundreds of μm and to cover the bacterial suspension to avoid drying or evaporation (shown in Fig 2 (A)).

The detailed procedure was as follows: The original *S*. *aureus* or *E*. *coli* suspensions, which were adjusted to McFarland Standard1.0, were diluted 1,000 times with phosphate-buffered saline (PBS) to a final bacterial density ranging from 2.5×10^5^ to 10×10^5^ cfu/ml. The sample film was wiped three times with an ethanol-impregnated paper cloth and set in a petri dish. Four hundred μl of the diluted solution was dropped onto a sample film and covered with a PET film of 25 cm^2^ in size. After incubation for 24 hr in an incubator at 37°C and 100% humidity, the medium consisting of 9.6 ml of fresh soybean-casein digest broth with lecithin and polysorbate 80 (SCDLP; Wako Pure Chemical Industries Ltd., Osaka, Japan) was poured directly onto the sample film in the petri dish, and the bacteria attached to the film surface were washed out by shaking the petri dish for 30 sec at 500 rpm/min using a vortex mixer. The bacterial suspension was then serially 10-fold diluted with PBS. One ml of each diluted sample was quickly mixed with 9 ml of melted Pearl Core plate count agar (PC; Eiken Chemical Co. Ltd., Tokyo, Japan) kept at 50°C in a water bath and poured onto a count agar plate. After incubation for 24 hr at 37°C, the number of colonies was counted.

#### ii) Droplet model

This method used a model designed to represent its practical application. A cross-sectional view during the test is shown in Fig 2 (B). The detailed procedure was as follows: The original *S*. *aureus* or *E*. *coli* suspension was diluted 100 times with 1/500NB to a final bacterial density ranging from 2.5×10^6^ to 10×10^6^ cfu/ml. Ten μl of the diluted solution was dropped onto a sample film, disinfected with ethanol, and kept in a safety cabinet at room temperature for the indicated time. The number of live bacteria on the sample film was then counted using the same method as that described above for the film covering method.

### Identification of antibacterial component(s)

To identify the antibacterial soluble component(s) in the resin, the films were immersed in water for 6 to 48 hr at 37°C. The influence of the elution of the effective components on antibacterial activity was then investigated by the covering method using *S*. *aureus*.

Identification of antibacterial component(s) in the elution water was carried out with time-of-flight secondary ion mass spectrometry (TOF-SIMS) and Fourier transform infrared spectroscopy (FT-IR) as follows.

#### i) Time of Flight Secondary Ion Mass Spectrometry (TOF-SIMS)

A 25 cm^2^ piece of Sample A film in a glass petri dish was immersed in 10ml of deionized water at 37°C for 24 hr with the resin side up. Ten μl of the water containing the components eluted from the resin was dropped on a silicon wafer and evaporated at 100°C for 10 min. The evaporation residue on the silicon wafer was analyzed by TOF-SIMS using TRIFT V nano TOF (Ulvac-Phi, Kanagawa, Japan), which is a technique used to obtain information regarding elements or molecular species within 1 nm of the sample surface through the detection of secondary ions emitted from the surface by irradiation with an ion beam (primary ion). The silicon wafer was used as the substrate for measurement to specify the background signal as Si.

#### ii) Fourier Transform Infrared spectroscopy (FT-IR)

Evaporation residue, prepared in the same manner as described above, was analyzed by FT-IR using Nicolet Avatar 370 (Thermo Fisher Scientific, Kanagawa, Japan), which is a technique used to obtain information regarding molecular structures and chemical bonds through the measurement of the absorbance of infrared light. The urethane acrylate monomer coating on the silicon wafer, which is the major component of the resin, was also analyzed for reference.

### Wettability test

Evaluation was carried out on the high wettability of the moth-eye film, which is associated with its distinctive super-hydrophilicity. The diameter of droplets immediately after application and their drying times at 24°C and 45% humidity were measured using three sample films each for Sample A, B and C.

### Preparation of [^3^H]-thymidine-labeled *S*. *aureus*

*S*. *aureus* was cultured in 1 ml of NB for 6 hr and pulsed with 10 μCi of 6-[^3^H]-thymidine (Perkin Elmer, Waltham, MA) for 30 min at 37°C. Labeled bacterial cells were collected by centrifugation and resuspended in one culture volume of 1/500 NB. After 100-fold dilution, samples were subjected to antibacterial assay using the droplet model. Radioactivity remained on the film was measured using a liquid scintillation counter LSC-6100 (Hitachi-Aloka Medical, Tokyo, Japan)

### Durability tests

Resistance of the moth-eye film to physical stress was evaluated after repetitive wiping with an ethanol-impregnated paper cloth by measurement of the remaining antibacterial activity using the droplet method.

### Scanning electron microscope (SEM) observation

Microscopic analysis was carried out using a S-4700 field emission scanning electron microscope (Hitachi High-Technologies, Tokyo, Japan). The procedure was as follows: Sample films were cut into 49 cm^2^ and 10 μl of *S*. *aureus* suspension, prepared according to the procedure described above, was dropped onto the film and dried without a cover in an incubator at 37°C for 1 hr. Sample films were rinsed with PBS to remove dirt, and fixed with 2.5% glutaraldehyde and paraformaldehyde for 1 hr to suppress chemical reactions due to bacterial death. Sample films underwent dewatering with gradually increasing ethyl-alcohol concentrations and freeze drying with tertiary-butyl alcohol. The detailed dewatering process was as follows: Rinsed sample films were immersed in 40% concentration ethanol for 5 min. This operation was repeated at ethanol concentrations of 60%, 70%, 80%, 90%, 95%, 100% and again at 100%. Then, replacement from ethanol to tertiary-butyl alcohol was conducted 4 times in the same manner at concentrations of 30%, 50%, 70%, 100% and again at 100%. Lastly, the sample films were fixed on the observation base with conductive tape and a 5-nm osmium film was deposited by sputtering.

An additional quantitative analysis of the distribution of bacterial bunch size was carried out for Sample A and B. The procedure was as follows: The observation elevation angle was set at 45 degrees and magnification at x2000. Ten images were taken at arbitrary points and the number of bacteria was counted in each bunch.

## Results

### Antibacterial effect of the moth-eye film

**Fig 3:**
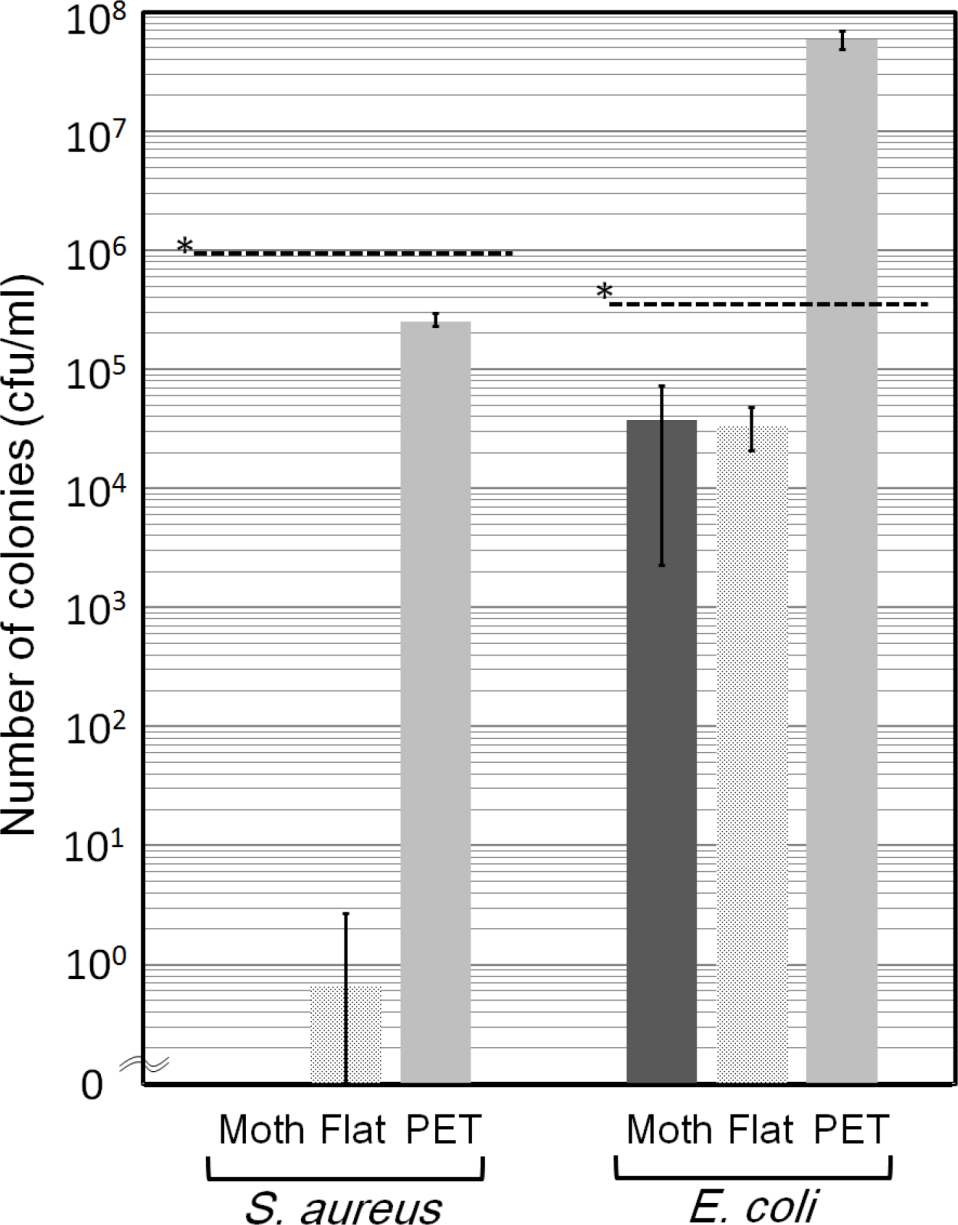
Antibacterial effect of the sample film against *S*. *aureus* and *E*. *coli* as evaluated using film covering method. The counts of viable bacteria on Sample A (Moth), Sample B (Flat) and Sample C (PET) in suspension after incubation for 24 hr. The dotted line denoted by * shows the initial number of bacteria. Error bars show the standard deviation values for sextuplicate measurements (Moth) and triplicate measurements (Flat and PET).

The antibacterial effect of the moth-eye film was first evaluated by the JIS Z2801 method, which is based on the logarithmic number of live bacteria after 24 hr-incubation using a covering film to keep the thickness of the bacterial suspension constant without evaporation (Fig 3). With regard to *S*. *aureus*, both Sample A and B showed significant antibacterial effects, with no colony-forming bacteria observed on Sample A, in particular. This indicated that some substance(s) in the resin predominantly showed disinfectant activity and/or an inhibitory effect on the replication of *S*. *aureus*. On the other hand, weak inhibitory effect was observed against *E*. *coli* using both Sample A and B.

### Analysis of the antibacterial component(s) in the resin

**Fig 4:**
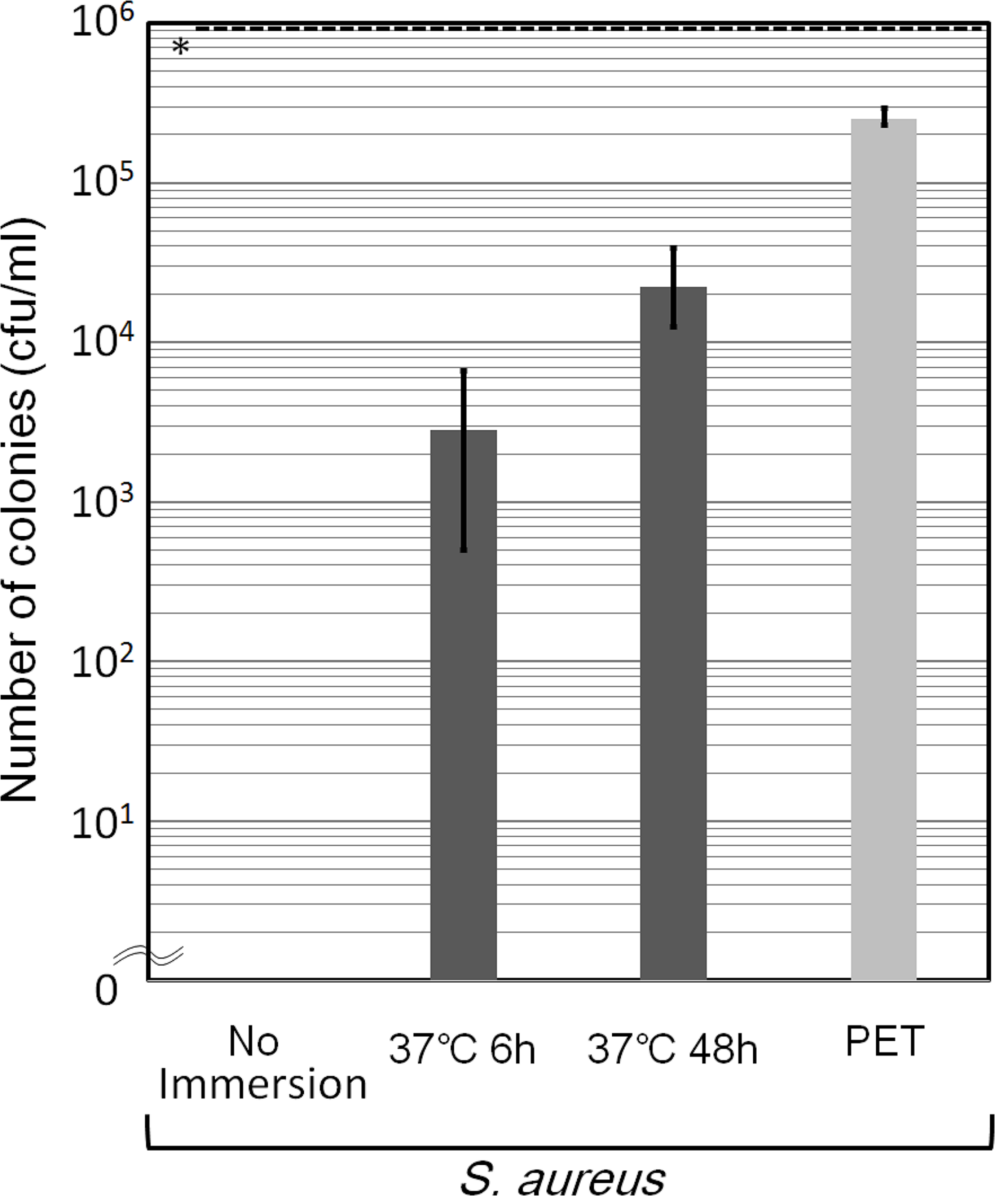
Antibacterial effect of moth-eye film against *S*. *aureus* after long-term immersion in water. The counts of viable bacteria on Sample A (Moth) without immersion, after 6 hr immersion, after 48 hr immersion, and those on Sample C (PET) after 24 hr incubation. The dotted line denoted by * shows the initial concentration of CFU. Error bars show the standard deviation values for triplicate measurements.

To determine the antibacterial substance(s) in the resin layer, the influence of long-term immersion of the moth-eye films in water against on the antibacterial effect was examined (Fig 4). The antibacterial activity of Sample A decreased significantly dependent on immersion time. The same test was preformed against Sample B and a similar result in terms of the loss of antibacterial activity was confirmed.

**Fig 5:**
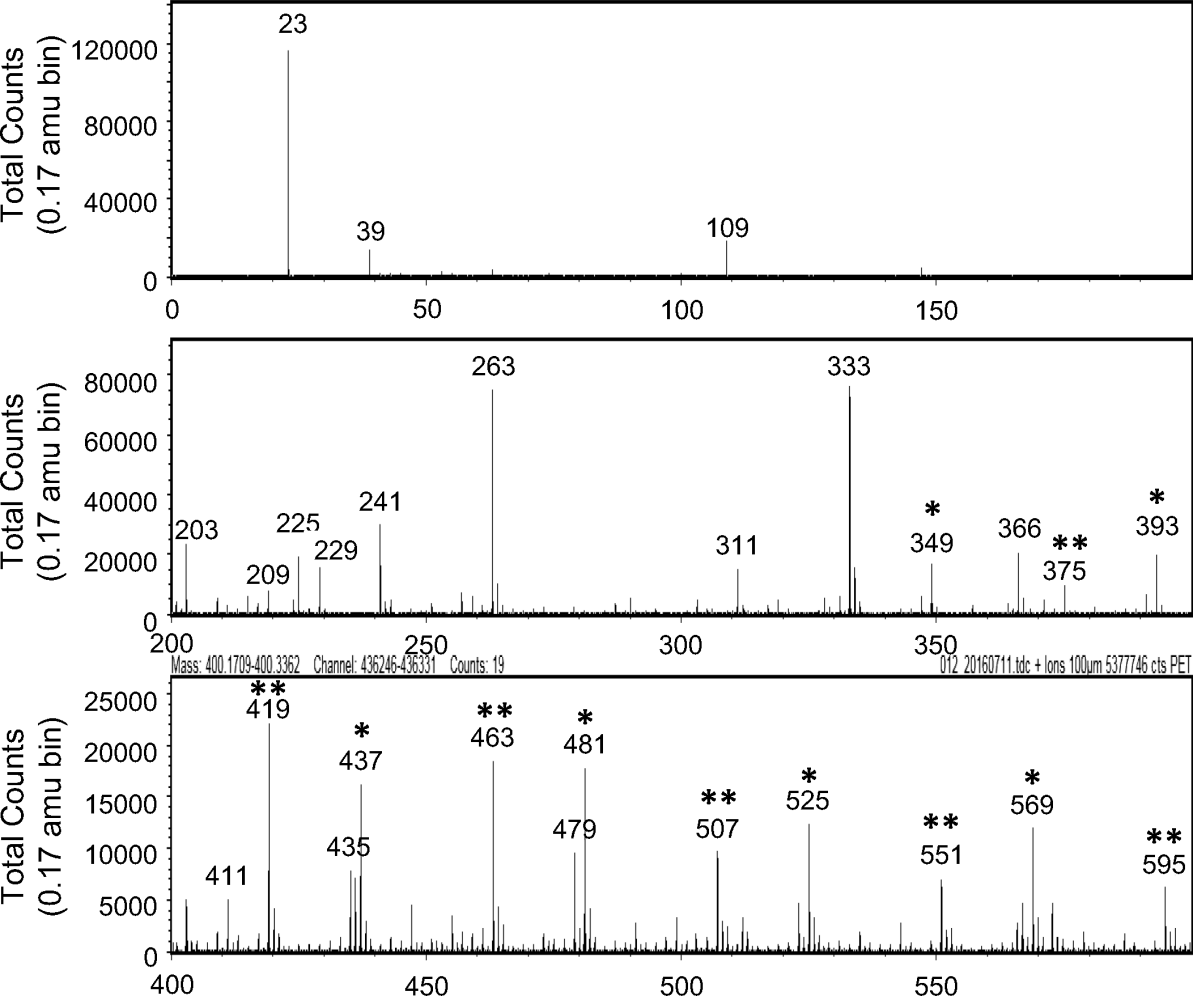
Time-of-flight secondary ion mass spectrometry (TOF-SIMS) spectra. Measurement of the eluted components from the super-hydrophilic moth-eye film obtained by immersion at 37°C for 24 hr in sterilized water.

**Fig 6:**
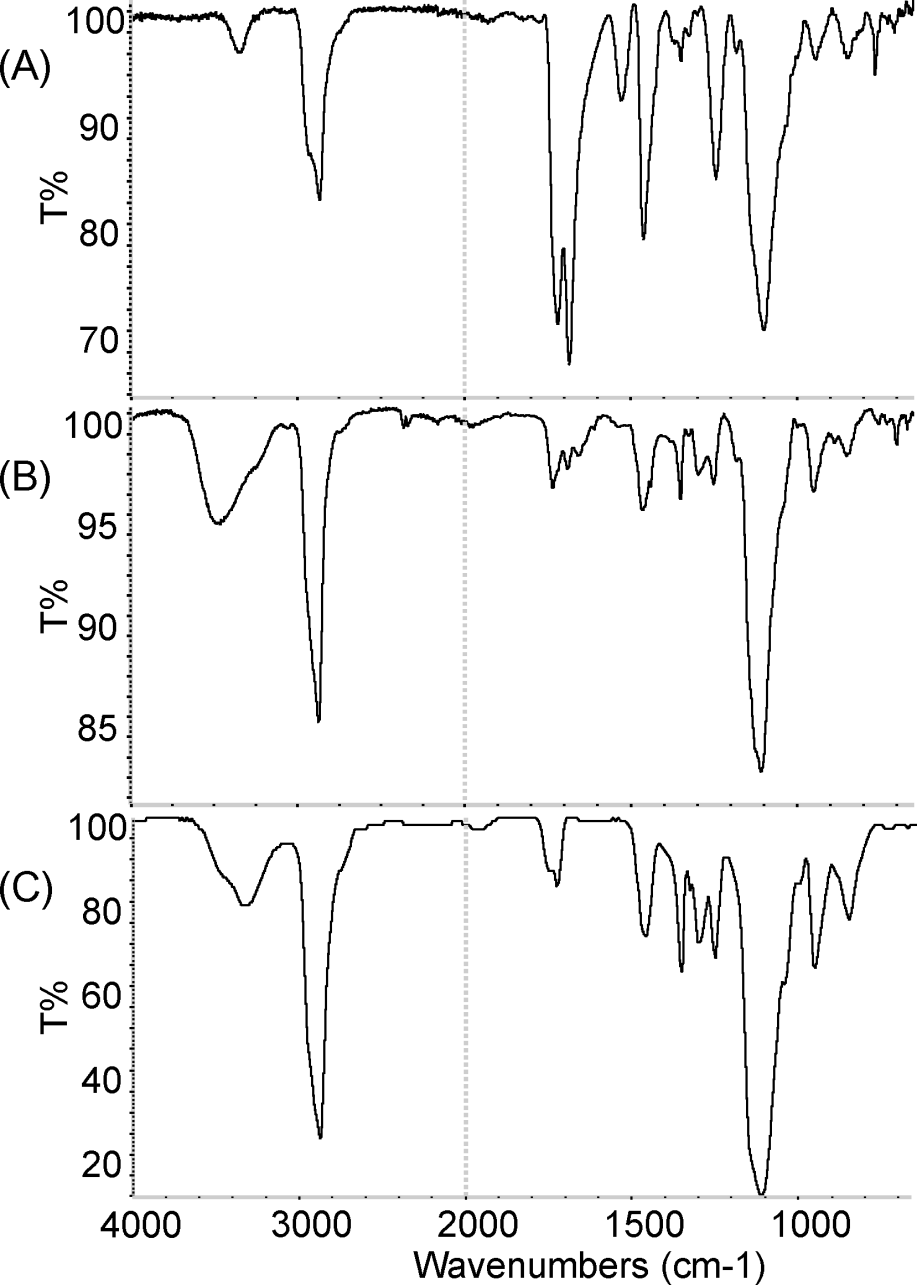
Fourier transform infrared spectroscopy (FT-IR) spectra. Measured spectra of the major single component of the resin containing urethane acrylate monomer with PEG derivatives (A) and eluted components from the super-hydrophilic moth-eye film (B) and reference PEG (C).

This result indicated that any soluble antibacterial substance(s) was contained in the resin layer and was readily eluted in water. To identify the substance(s), the elution water was analyzed by TOF-SIMS. Interestingly, two series of peaks, both composed of 6 peaks with 44 interval values, were detected (indicated by * and ** in Fig 5). These residues are thought to be composed of PEG derivatives, as the value corresponded to the molecular weight of a single unit of the PEG chain, C_2_H_4_O. The two detected series also suggested that the PEG derivatives were composed of two types of materials having different derivatives as the terminal group of the PEG derivatives. To identify the eluted component(s), urethane acrylate monomer with PEG derivatives, the residue eluted from the moth-eye film, and data from a PEG library were comparatively analyzed by FT-IR (Fig 6). The results showed that the spectrum of the residue eluted from the moth-eye film was in better agreement with that of PEG derivatives than that of urethane acrylate monomer, supporting the results of the TOF-SIMS.

### Antibacterial effect of the moth-eye film evaluated using the droplet method

One characteristics of the moth-eye film made from hydrophilic urethane acrylate is its hydrophobicity. A small volume of water dropped on the surface spread and dried quickly. To clarify the merits of this feature for antibacterial materials, we studied the effect on the moth-eye film using a droplet model.

**Fig 7:**
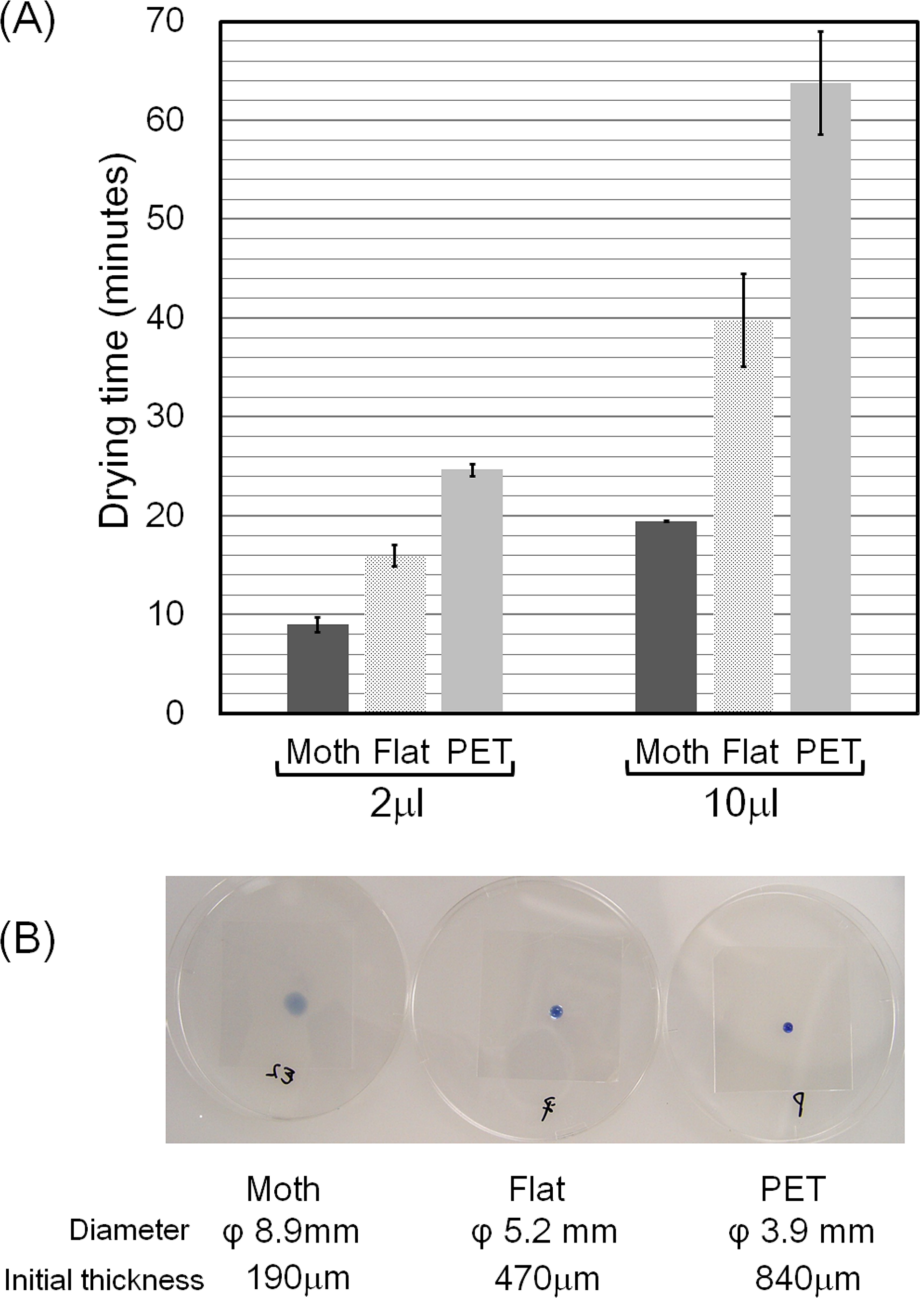
Drying time of droplets on the sample films. (A) Sample A (Moth), Sample B (Flat), and Sample C (PET). (B) Top views of 10 μl droplets of giemsa stain solution applied to 25 cm^2^ film samples. Error bars show the standard deviation values for duplicate measurements.

The relative drying time of Sample A was 2.2 and 3.3 times shorter than that for Sample C for droplets of 2 and 10 μl in volume, respectively (Fig 7(A)). Similarly, that of Sample A was 1.7 and 2.0 times shorter than that for Sample B for droplets of 2 and 10 μl in volume, respectively. Regarding the spreading property, the diameters of the 10 μl droplets of giemsa stain solution immediately after application were 8.9, 5.2 and 3.9 mm for Sample A, B and C, respectively (Fig 7(B)). Overall, it was found that Sample A has extremely quick spreading and drying properties.

**Fig 8:**
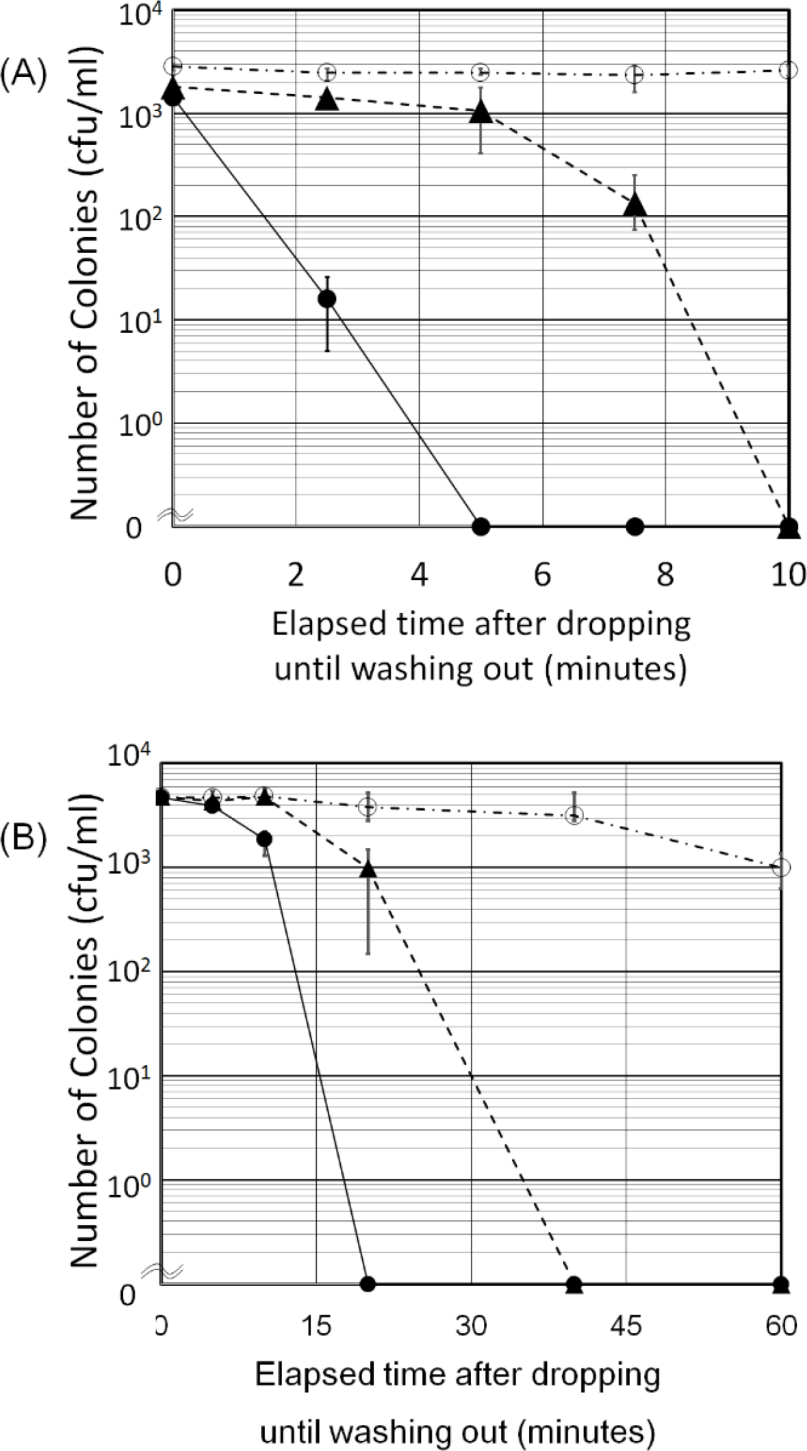
Antibacterial effects evaluated using the one droplet method. (A) The counts of viable *S*. *aureus* (A) and *E*. *coli* (B) bacteria on the sample films. ●: Sample A (Moth), ▲: Sample B (Flat), ○: Sample C (PET). Error bars show the standard deviation values for triplicate measurements.

The antibacterial activity of the moth-eye film against bacteria in a droplet was estimated as a model of its practical application. For *S*. *aureus*, the antibacterial effect of Sample A was 100 times greater at 2.5 min and 1000 times greater from 5 to 7.5 min than those of Sample B, as shown in Fig 8 (A). As for *E*. *coli*, the effect appeared after the sample films dried, which is in contrast with the results of the experiment using *S*. *aureus*. The antibacterial effect of Sample A was 1000 times greater at 20 min than that of Sample B, as shown in Fig 8 (B). Interestingly, 10% of the *E*. *coli* on Sample C survived for 120 min after the droplets of bacterial solution were applied and the solvent water had evaporated completely, although *E*. *coli* on the moth-eye films could not survive for even 20 min.

**Fig 9:**
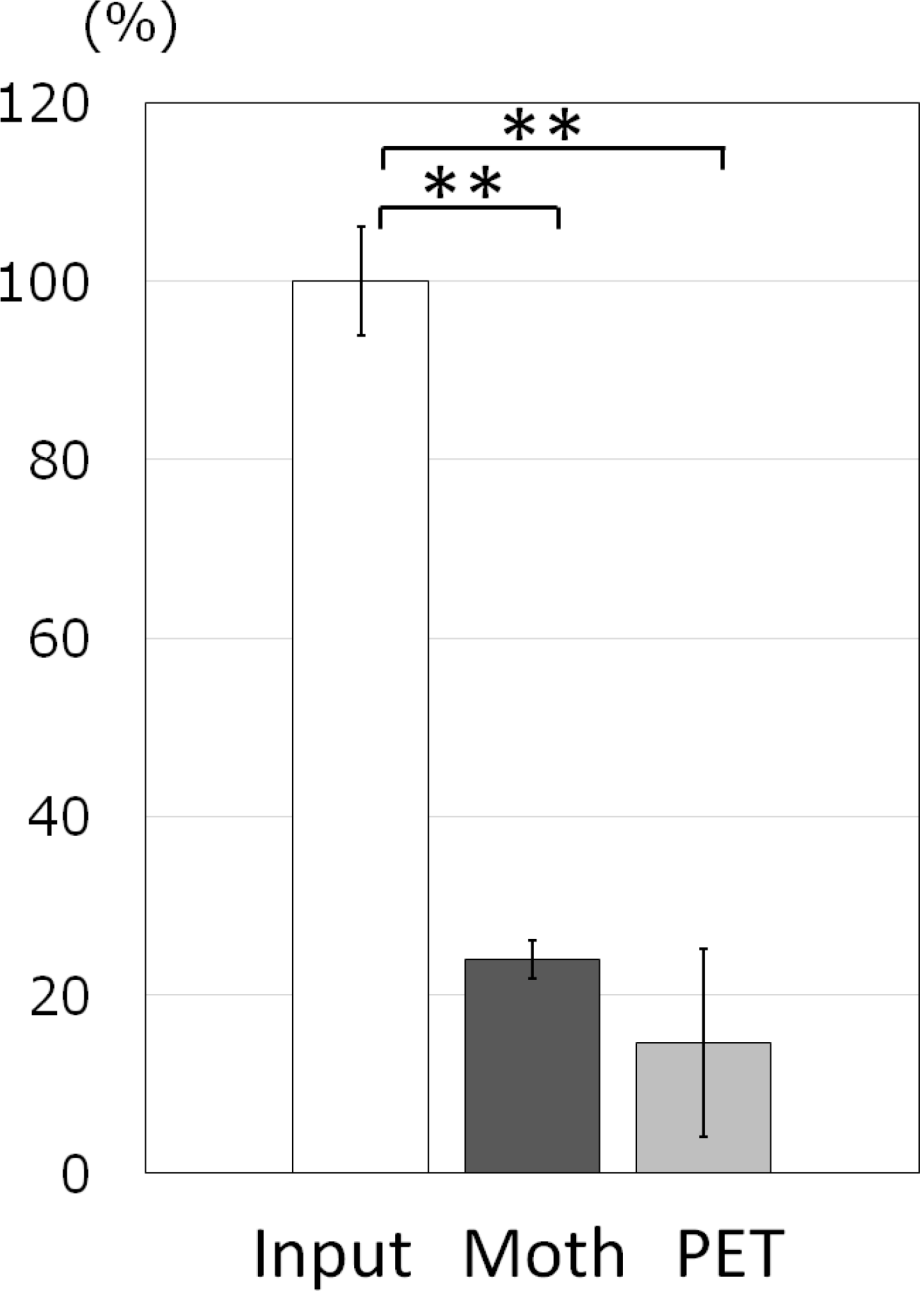
The ratios of residual [^3^H]-Thymidine-labeled *S*. *aureus* on moth-eye and PET films. A diluted solution of [^3^H]-Thymidine-incorporated *S*. *aureus* (Input) was spotted on the moth-eye and PET films. After washing, as described in Materials and Methods, the counts of residual [^3^H] on these films were analyzed by liquid scintillation. Data are representative of three independent experiments. Values expressed are the mean ± SE for triplicate measurements. **p<0.01 (versus time zero by Student’s t-test).

In order to exclude the possibility that the rapid decrease in culturable bacteria on Sample A described above resulted from the firm attachment of the bacteria on the film surface allowing them to remain on the film despite washing, the ratio of residual bacteria on the film was measured using [^3^H]-labeled *S*. *aureus* (Fig 9). The results showed that about 75% of the bacteria were washed out from the film, confirming that the *S*. *aureus* were actually killed on moth-eye film in the droplet model and did not simply remain attached on the film surface.

**Fig 10:**
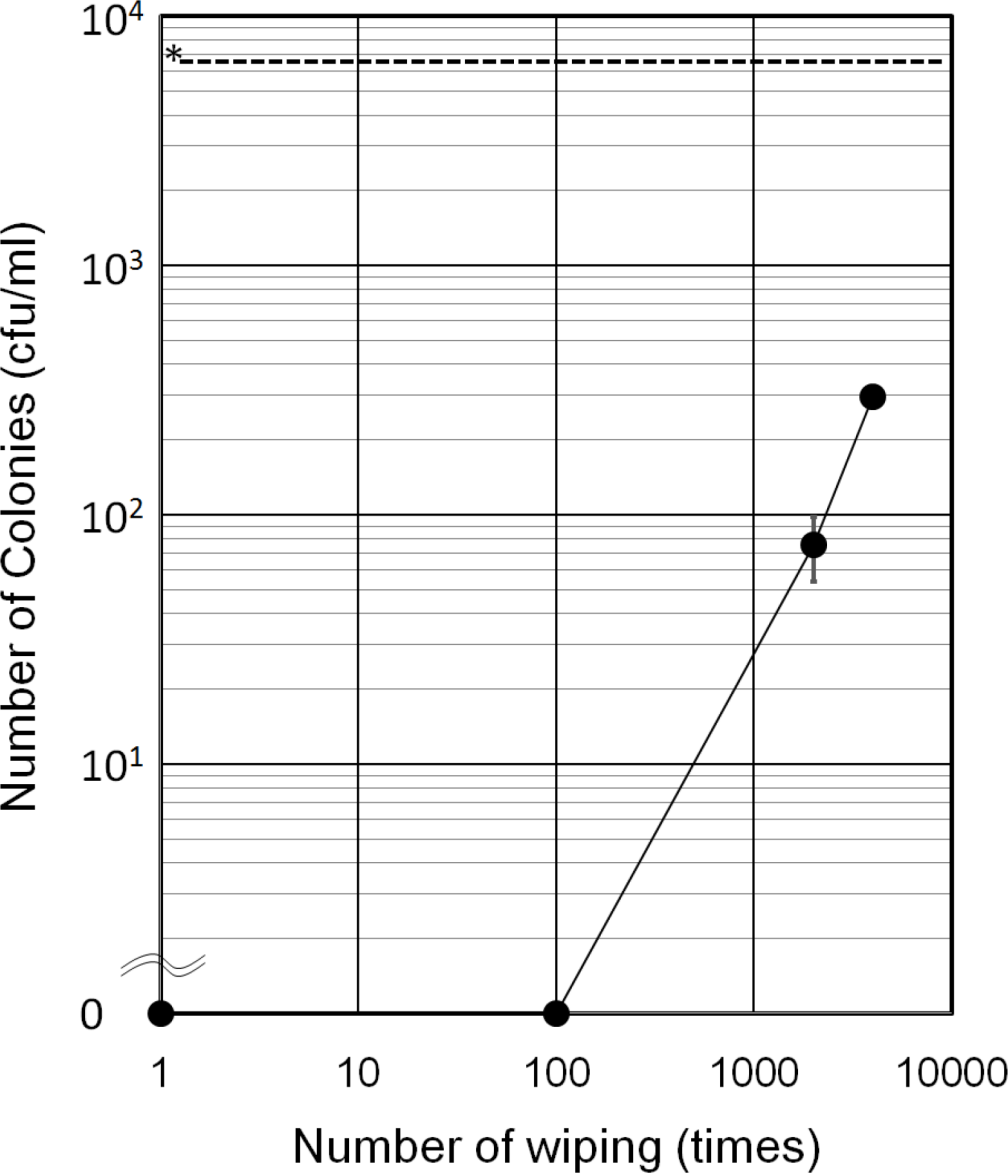
Antibacterial effect of the moth-eye film after repetitive wiping. The dotted line denoted by * shows the initial concentration of CFU. Error bars show the standard deviation values for duplicate measurements.

To estimate the durability of the moth-eye film, sample films were wiped with an ethanol-impregnated paper cloth up to 4000 times and the residual antibacterial effect was evaluated using the droplet method with *S*. *aureus* (Fig 10). It was found that the antibacterial effect was not degraded at all by wiping 100 times and was only gradually degraded by wiping over 2000 times, although the bacterial numbers decreased to one 86th and one 22nd after wiping 2000 and 4000 times, respectively. It was confirmed by SEM observation that the moth-eye structure was remained. These results indicated that the effective antibacterial components, considered to be PEG derivative, were removed by wiping with an ethanol-impregnated paper cloth, as was observed on immersion in sterilized water.

### Scanning electron microscope (SEM) observation

**Fig 11:**
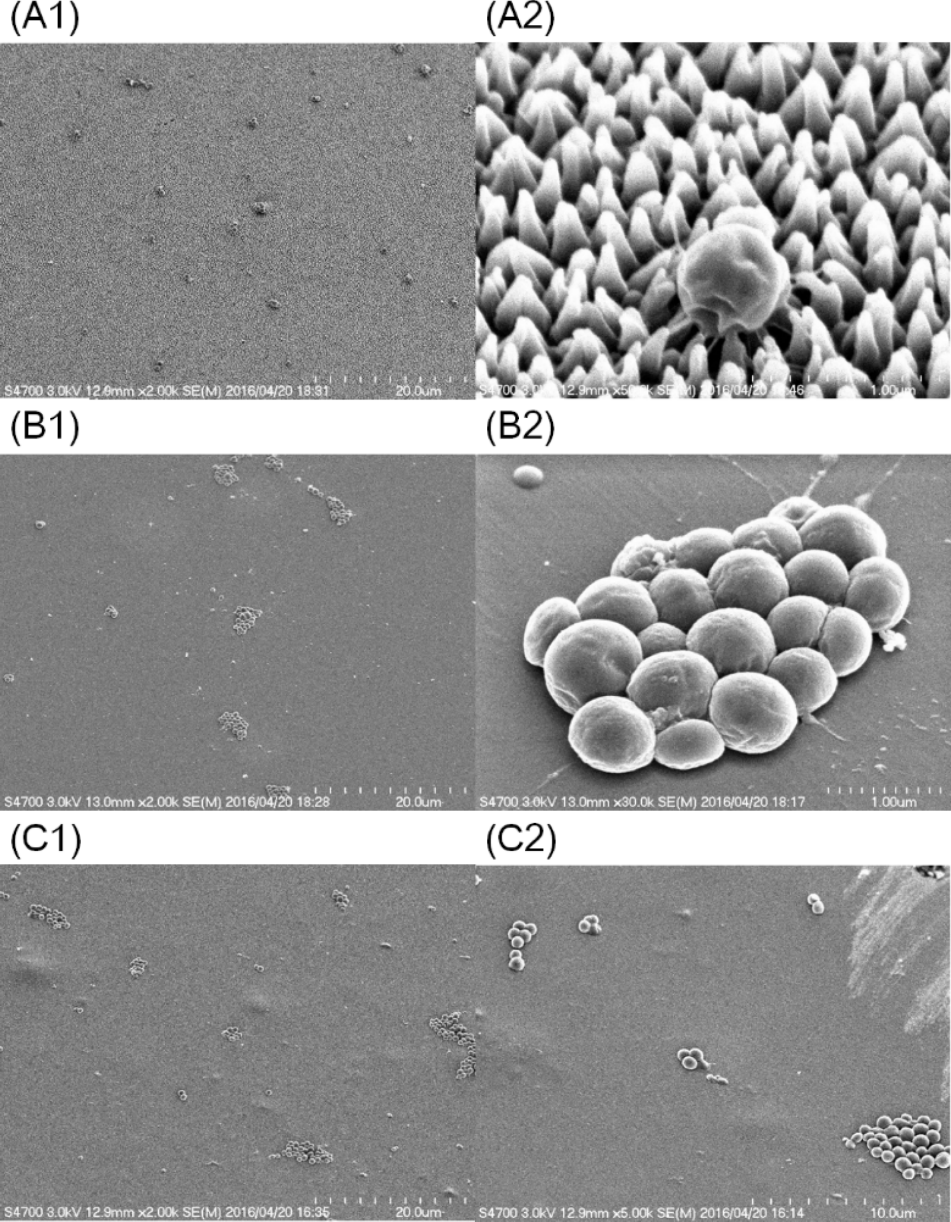
Representative SEM micrographs of bacteria on sample films. (A1) and (A2) Sample A (Moth), (B1) and (B2) Sample B (Flat), and (C1) and (C2) Sample C (PET). Magnifications are x2000 for A1, B1 and C1, x50,000 for A2, x30,000 for B2 and x5000 for C2. All images were taken at 3 kV.

For morphological observation of the phenomena described above, scanning electron macroscopy (SEM) were carried out. It was remarkable that many of the observed bacteria formed relatively small bunches composed of 5 or less single cells on Sample A, whereas relatively large bunches were observed on Sample B and C (Fig 11).

Distribution of bacterial bunch size was also analyzed (Table 1). On Sample A, the ratio of small bunches composed of less than 5 cells was larger than that on Sample B. It appeared that the rapid diffusion of the buffer on the moth-eye film forced the *S*. *aureus* to disperse and make tight contact with the surface of the film.

**Table 1:** Analysis of bacteria bunch size observed on the films.

	Ratio of cell numbers in each colony to total cells by colony size			
	From 1 to 5 cells	From 6 to 10 cells	From 11 to 20 cells	Over 20 cells
**Sample A (Moth)**	**54%**	**25%**	**8%**	**13%**
**Sample B (Flat)**	**10%**	**6%**	**23%**	**62%**

## Discussion

### Effects of the chemical components in the hydrophilic resin

Significant differences in antibacterial effect, in that the live bacteria in the titers decreased by more than three logs, have been confirmed between PET films (Sample C) and hydrophilic resin-coated films in which the resin was composed of hydrophilic urethane acrylate having PEG derivatives as spacers (Sample A and B) by both the film covering (Fig 3) method and the droplet method (Fig 8). The origin of the antibacterial effect observed for the film covering method can be attributed to specific chemical components in the resin currently used, not to the moth-eye nanostructure, as the effects of the moth-eye films and flat films were similar on the basis of the experimental results (Fig 3). In addition, immersion in sterilized water (Fig 4) and wiping with an ethanol-impregnated paper cloth (Fig 10) both also indicated that some ingredient in the resin contributed to the antibacterial property. This represents the feature of this technology. These experimental results are in agreement with those from a report showing that nano-pillars alone do not have any antibacterial property [14].

TOF-SIMS and FT-IR analyses showed that the effective ingredient was considered to be some component(s) included in the PEG derivatives, not PEG alone, as PEG is not commonly known to possess antibacterial activity. FT-IR results suggest that the residue eluted from the resin contains PEG chains modified with some derivatives, since the spectrum of the residue eluted from the resin was not fully in agreement with the data from a PEG library. TOF-SIMS results showed that there were two kinds of derivatives, not one. Interactions between the bacteria and one or both derivatives can be considered to play a role in bestowing the antibacterial activity. Further, the results suggest that the resin interacted more strongly against *S*. *aureus* than against *E*. *coli* (Fig 3 and Fig 8). Taking the strength of the outer membranes into account, we believe that the antibacterial effect against Gram-negative *E*. *coli* should be exhibited earlier than that against Gram-positive *S*. *aureus* due to the thickness and structure of the cell wall, although the opposite results were obtained. The adhesiveness between the outer membrane of *S*. *aureus* and the material including the PEG chains with the two derivatives might be stronger than that of *E*. *coli*.

### Effects of the nano structure on the surface of the moth-eye films

The film covering method is not suitable for evaluating the practical usage of antibacterial films in terms of maintaining the bacteria suspension at a constant thickness of a few hundreds of μm, covering the bacterial suspension to avoid drying or evaporation, and incubating for 24 hr at 37°C and 100% humidity. The first two reasons, in particular, are strongly dependent on film properties. For example, the quick drying of water containing bacteria is commonly considered to suppress bacterial growth. This characteristic intrinsically possessed by the film needs to be regarded as part of the total antibacterial property of the sample films. Our proposed droplet model is, therefore, a better means of evaluating antibacterial activity in this respect.

With regard to the geometric properties of nano-pillars, the results of the droplet method indicated that the moth-eye films are effective against *S*. *aureus* and *E*. *coli*. There were also significant differences in elapsed time between Sample A and B; 5 min for *S*. *aureus* (Fig 8(A)) and 20 min for *E*. *coli* (Fig 8(B)). The chemical properties of the resin and the physical properties of the nanostructure are considered to produce a synergetic antibacterial effect. Left shifts from the dashed line ▲ to the solid line ● in Fig 8 correspond to the physical properties of the nanostructure.

Macroscopically, the hydrophilic moth-eye film (Sample A) and hydrophilic flat-surfaced film (Sample B) exhibited a marked difference in drying time and area of spread immediately after application of the bacterial suspension. The super-hydrophilic moth-eye film allows the droplets of bacterial suspension to spread readily, enlarge its surface area, and this led to a significantly shorter drying time. It is commonly known that bacteria cannot grow or survive long under dry conditions. This represents the second feature of this technology, which is due to distinctive super-hydrophilicity caused by the moth-eye film nanostructure.

The assumed antibacterial mechanism of the moth-eye films can be explained as follows. The enlargement of the surface area corresponded to a decrease in the thickness of the droplet. The thinner the droplet, the higher was the chance of the bacteria coming into contact with the surface (Fig 7(B)). Once the bacteria came into contact with the surface, they were trapped with the assistance of the adhesive property of the PEG derivatives and killed. Based on this proposed mechanism, the hydrophilic flat surface films would also have an antibacterial effect, but would be less effective than the hydrophilic moth-eye films. This represents the third feature of this technology. Accordingly, one of the origins of the antibacterial property observed in the moth-eye films could be the localization of the PEG chains with some derivatives on the surface of the film, which induces the bacteria to adhere to the surface. Based on this notion, it appears that PEG chains with some derivatives should not be eluted, but preserved on the surface to maintain the antibacterial effect. Unfortunately, the current resin is not resistant to elution at present, and further development is necessary to improve the water resistance of the current resin.

Confirmed experimental results regarding the superiority and effectiveness of nano-pillars are limited to their rapid drying and spreading properties at the moment. However, the results of this study suggested that the nano-sized structures of the moth-eye films contributed to their antibacterial activity.

SEM observation revealed other microscopic aspects of the antimicrobial effect in that the moth-eye films suppressed the attachment of bacterial bunches. The results were considered to be derived from the extremely high wettability of the moth-eye films, which induced a strong flows to disperse the bacteria from bunches into individual cells. The antibacterial effect of the hydrophilic moth-eye films is also expected to depend on this aspect, as *S*. *aureus* exhibit a pathogenic property when formed into bunches but are less pathogenic when existing as single cells [22]. In addition, it was found from SEM observations (Fig 10(A2)) that a single cell of *S*. *aureus* secreted a number of microfibers. The microfibers are considered to be involved in the interactions between the bacteria and the resin on the basis of the observational results that revealed that the microfibers were more distinctively observed on moth-eye films than on other films.

Lastly, examinations of the antibacterial effect against influenza virus have been undertaken using the film covering method, but no positive results have yet been obtained. This might be due to differences in size between the virus and the nano-pillars and/or the adhesion between the outer membrane, envelope, and resin surface does not act to kill the virus effectively.

## Conclusions

The artificial surfaces of nanoimprinted moth-eye films have been confirmed to exhibit significant and immediate antibacterial effect under dry conditions at room temperature and normal humidity. It was found that the antibacterial effect of the moth-eye films was derived from the synergetic effect of the chemical properties of the resin and the physical properties of the nanostructure.

The benefit of this synergetic effect was immediate the antibacterial effect, and it was considered that this antibacterial approach based on the use of moth-eye films could provide one of the most promising candidates for infection inhibition in sanitation-conscious facilities such as hospitals and food-processing factories.
